# A Phase Ib trial of CA4P (combretastatin A-4 phosphate), carboplatin, and paclitaxel in patients with advanced cancer

**DOI:** 10.1038/sj.bjc.6605650

**Published:** 2010-04-13

**Authors:** G J Rustin, G Shreeves, P D Nathan, A Gaya, T S Ganesan, D Wang, J Boxall, L Poupard, D J Chaplin, M R L Stratford, J Balkissoon, M Zweifel

**Affiliations:** 1 Department of Medical Oncology, Mount Vernon Cancer Centre, Northwood, Middlesex, UK; 2 The Churchill Hospital, Headington, Oxford, UK; 3 Josephine Ford Cancer Center/Henry Ford Health System, Detroit, MI, USA; 4 OXiGENE Inc., San Francisco, CA, USA; 5 Gray Institute for Radiation Oncology and Biology, University of Oxford, Oxford, UK

**Keywords:** CA4P, carboplatin, combretastatin A4 phosphate, paclitaxel, phase I trial, vascular disrupting agent

## Abstract

**Background:**

The vascular disrupting agent combretastatin A4 phosphate (CA4P) causes major regression of animal tumours when given as combination therapy.

**Methods:**

Patients with advanced cancer refractory to standard therapy were treated with CA4P as a 10-min infusion, 20 h before carboplatin, paclitaxel, or paclitaxel, followed by carboplatin.

**Results:**

Combretastatin A4 phosphate was escalated from 36 to 54 mg m^−2^ with the carboplatin area under the concentration curve (AUC) 4–5, from 27 to 54 mg m^−2^ with paclitaxel 135–175 mg m^−2^, and from 54 to 72 mg m^−2^ with carboplatin AUC 5 and paclitaxel 175 mg m^−2^. Grade 3 or 4 neutropenia was seen in 17%, and thrombocytopenia only in 4% of 46 patients. Grade 1–3 hypertension (26% of patients) and grade 1–3 tumour pain (65% of patients) were the most typical non-haematological toxicities. Dose-limiting toxicity of grade 3 hypertension or grade 3 ataxia was seen in two patients at 72 mg m^−2^. Responses were seen in 10 of 46 (22%) patients with ovarian, oesophageal, small-cell lung cancer, and melanoma.

**Conclusion:**

The combination of CA4P with carboplatin and paclitaxel was well tolerated in the majority of patients with adequate premedication and had antitumour activity in patients who were heavily pretreated.

Tumour vascular disrupting agents (VDAs) are a new class of cancer therapies that target the existing vasculature of tumours to cause rapid vascular shutdown in the tumour, leading to cell death (Patterson and Rustin, 2007). Combretastatin A4 Phosphate (CA4P) is a tubulin-binding VDA that displays potent and selective toxicity towards tumour vasculature (Tozer *et al*, 1999). After single-agent administration in preclinical models, rapid regrowth of the viable tumour rim is observed, which obtains its nutrients from the surrounding normal tissue and blood supply (Salmon and Siemann, 2007). However, when CA4P is combined with a variety of cytotoxic agents, as well as with radiotherapy and antiangiogenesis inhibitors, enhanced tumour control is achieved *in vivo* (Grosios *et al*, 2000; Murata *et al*, 2001; Siemann *et al*, 2002; Staflin *et al*, 2006; Yeung *et al*, 2007).

Four phase 1 trials of CA4P have demonstrated minimal single-agent antitumour activity, and have confirmed that it has vascular disrupting activity according to functional imaging (Dowlati *et al*, 2002; Stevenson *et al*, 2003; Rustin *et al*, 2003a; Cooney *et al*, 2006). Dose-limiting toxicities (DLTs) were seen at doses above 50 mg m^−2^. In view of the enhanced activity observed when CA4P is combined with cisplatin (El Zayat *et al*, 1993), carboplatin (Landuyt *et al*, 2000), CPT11 (Wildiers *et al*, 2004), or paclitaxel (Yeung *et al*, 2007) *in vivo*, human combination studies are the next step.

A phase I trial of carboplatin (dose to produce an area under the concentration curve (AUC) 4 or 5), followed 1 h later by CA4P 27–36 mg m^−2^, demonstrated grade 3 or 4 thrombocytopenia in 9 of 16 (56%) patients in any course (Bilenker *et al*, 2005). Decreased renal perfusion was likely the cause of reduced carboplatin clearance and subsequent increase in myelotoxicity (Anderson *et al*, 2003). This suggests that when administering CA4P in combination with carboplatin, the reduction in renal perfusion induced by CA4P needs to have recovered before administering carboplatin. Administering carboplatin/paclitaxel 24 h after CA4P has been shown to be as effective as administering it before CA4P in MDA MB-231 breast adenocarcinoma xenografts (Edvardsen and Chaplin, personal communication). There is evidence that 24 h after CA4P administration, there is both continued proliferation of the viable tumour rim and revascularisation, the latter stimulated at least in part by circulating endothelial progenitor cells (Shaked *et al*, 2006). This might therefore be the best time to administer carboplatin. In clinical practice, carboplatin is frequently given in combination with paclitaxel for lung and ovarian cancer. As this combination showed enhanced activity when administered with CA4P in the KAT-4 model (nude mice bearing human anaplastic thyroid cancer xenografts) (Yeung *et al*, 2007) and as better therapies are needed for these cancers, a clinical trial was indicated. In view of the toxicity observed by Bilenker *et al* (2005), we planned a dose-escalation and pharmacokinetic study initially combining CA4P with carboplatin or paclitaxel as doublets before combining all three drugs.

## Materials and methods

### Patient selection

Patients with histologically confirmed cancer, not amenable to standard therapy or refractory to conventional therapy, were eligible for this study. Other eligibility requirements included ECOG performance status 0–2; life expectancy ⩾4 months; age ⩾18 years; adequate bone marrow function (granulocyte count >1500 cells mm^−3^; platelet count >100 000 cells mm^−3^); adequate hepatic function (total bilirubin <1.5 mg per 100 ml; ALT and AST <2.5 × upper limit of normal); adequate renal function with glomerular filtration rate (GFR) measured by EDTA clearance >50 ml min^−1^; no other anticancer therapy for 4 weeks; no active concurrent malignancies except cone-biopsied *in situ* carcinoma of the cervix, or adequately treated basal or squamous carcinoma of skin.

Patients were excluded if they had brain metastases, serious infection or other non-malignant illness, ⩾grade 2 neuropathy, major surgery within 4 weeks, previously administered radical radiotherapy or evidence of vascular damage from radiotherapy, history of peripheral vascular disease, history of angina, myocardial infarction, arrhythmias, or conditions associated with QTc prolongation, uncontrolled hypertension, or anticoagulation apart from low-dose warfarin for maintenance of central line patency.

### Study design

This was a three-centre, open-label dose-escalation study to initially assess the safety and tolerability of combining CA4P at doses ranging from 36 to 60 mg m^−2^, with carboplatin doses from AUC 4 to 5, and of combining CA4P at doses from 27 to 54 mg m^−2^ with paclitaxel doses from 135 to 175 mg m^−2^. The next stage was combining CA4P with both carboplatin and paclitaxel. The actual dose-escalation scheme achieved is shown in the results section (Table 1). The study was approved by participating hospitals ethical review boards, and all enrolled patients provided written informed consent.

### Treatment and dose escalation

On day 1 of each 21-day cycle, patients received a 10-min infusion of CA4P. Routine premedication was not mandated but if toxicity occurred with the first course, premedication with dexamethasone and metaclopramide was suggested for future courses. On day 2, 18–22 h after administering CA4P, patients received a 60-min infusion of carboplatin or a 3-h infusion of paclitaxel. Glomerular filtration rate was measured by EDTA clearance. The dose of carboplatin corresponded to a target AUC and was calculated using a modified Calvert formula: 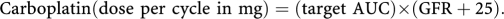


Provided no DLT occurred in any of the three patients per cohort, doses were increased as shown in Table 1. Once it had been ascertained that no DLTs were observed at a CA4P dose of 54 mg m^−2^ in either of the doublets, patients were then treated with the triplet of drugs at doses shown in Table 1. If a DLT was observed, three additional patients could be recruited to that dose level. The triplet consisted of CA4P on day 1, followed 18–22 h later by a 3-h infusion of paclitaxel, then a 60-min infusion of carboplatin. A dose-modification schedule based on grade ⩾2 neurological or cardiac toxicity and grade 3 or 4 other toxicities was adhered to.

Dose-limiting toxicity was defined as any of the following occurring in the first cycle: QTc prolongation ⩾500 ms, >grade 2 ventricular arrhythmia, grade 3 or 4 non-haematological toxicity (except fatigue/asthenia, nausea and/or vomiting), toxicity resulting in a treatment delay of >14 days, absolute granulocyte count <500 cells mm^−3^ for >5 consecutive days or febrile neutropenia with granulocyte count <1000 cells mm^−3^, thrombocytopenia <25 000 cells mm^−3^ or bleeding episode requiring platelet transfusion, grade ⩾2 neuropathy, which does not recover to grade 1 within 14 days after scheduled retreatment, or any grade toxicity requiring patient removal from the study on the basis of the judgement of investigators.

The maximum tolerated dose was defined as the maximum dose level of CA4P, administered in combination with carboplatin and paclitaxel, at which one or fewer patients experience a DLT.

### Treatment assessment

Laboratory assessments (including full blood counts) were performed weekly. Tumour evaluations were carried out at screening and then every two cycles. Criteria for response were based on Response Evaluation Criteria in Solid Tumours (RECIST) (Therasse *et al*, 2000) and response according to CA-125 was based on definitions agreed by the Gynaecologic Cancer Intergroup (Rustin, 2003).

### Pharmacokinetics

The plasma pharmacokinetics (PK) of carboplatin and paclitaxel were evaluated during cycle 1 in all patients receiving study treatment. For carboplatin and paclitaxel PK, blood samples were collected 20 h after CA4P infusion, on starting the 60-min carboplatin infusion and/or the 3-h paclitaxel infusion, respectively.

Plasma concentration data for paclitaxel and carboplatin plasma ultrafiltrate concentrations were analysed using non-compartmental methods. Peak concentrations (*C*_max_) were determined manually. The terminal elimination rate constants (*λ*_z_) were determined by linear regression analysis of the terminal log-linear part of the concentration–time curve. The total area under the observed plasma concentration–time curve (AUC) was calculated for each analyte from time zero to the last measured concentration, using the linear-log trapezoid rule. Area under the concentration curve values were extrapolated from the last observed time point to infinity by adding the last measured concentration divided by *λ*_z_.

### Statistical analysis

Non-parametric tests were used to calculate the statistical significance of differences between several groups (the Kruskal–Wallis test), and between two groups for paired (Wilcoxon signed rank-sum test) and unpaired (the Mann–Whitney *U*-test) data, and of correlations (Spearman).

## Results

### Patients and dose escalation

Between 27 June 2003 and 1 November 2005, 46 patients with a median age of 58 years were enrolled. Patient characteristics are listed in Table 2.

The dose-escalation schedule and the number of patients treated in each cohort are shown in Table 1. A total of 180 cycles were given to 46 patients, with 15 patients completing all 6 cycles. In all, 17 patients withdrew because of tumour progression, three because of early death (all due to tumour progression), five because of toxicity (two patients with grade 3 fatigue, one patient with grade 2 sensory neuropathy, one patient with a grade 4 allergic reaction to carboplatin, and one patient with grade 3 neutropenia and flu-like symptoms), and six for other reasons (one patient needed surgery for small-bowel obstruction, one patient experienced a clonic seizure, one patient was unwilling to continue, two patients had no clear clinical benefit, and the investigator decided to withdraw one patient). Of 180 cycles, 15 (8%) had to be delayed, mostly because of haematological toxicity.

### Haematological toxicity

The worst grade of haematological toxicity for each patient at each dose level is shown in Table 3. Despite blood counts being performed weekly, grade 3 or 4 thrombocytopenia was only seen in two patients: one in the paclitaxel doublet arm and one in the triplet arm. Grade 3 and 4 neutropenia was only seen in eight patients, all of them in the triplet arm.

### Non-haematological toxicity

Worst drug-related toxicities are summarised in Table 4. Overall, pain was one of the commonest toxicities and almost invariably an exacerbation of preexisting (tumour) pain, typically starting within 1 h of the commencement of the CA4P infusion and lasting up to a few hours, affecting 30 out of 46 patients (65%). No patient stopped treatment because of pain, as it was controllable with analgesia that included morphine. Neuropathy (paraesthesia) was more common in the paclitaxel (53%) and triplet (67%) arms, but there were still 47% of patients in the CA4P/carboplatin arm experiencing paraesthesia grade 1–3.

Rare but important non-haematological toxicities included allergic and neurological reactions. One patient experienced a non-fatal anaphylactic shock (common terminology criteria for adverse events (CTCAE) grade 4) as an allergic reaction to carboplatin. Short-lived and spontaneously resolving muscle weakness of the legs and arms (CTCAE grade 3 and 2) was observed in two patients, although the second patient's symptoms may also be explained by malignant meningeal infiltration, which was only diagnosed later.

Ataxia was seen in two patients (CTCAE grade 2 and 3) and resolved spontaneously within a couple of hours. CTCAE grade 3 ataxia occurred 1 day after the first CA4P infusion in one patient and was considered DLT.

Short-lived and spontaneously resolving dysphasia was observed in two patients treated in trial arm 1 at a CA4P dose of 54 mg m^−2^. One patient was unable to speak for 1 min, 2 h after his second CA4P infusion (initially deemed CTCAE grade 1 by the local investigator, amended to grade 2 upon monitoring as there is no grade 1 for such an event according to CTCAE Version 3.0), and a second patient experienced dysphasia the day after the first infusion of CA4P, lasting less than 5 min. This event was initially deemed grade 2 by the local investigator, but amended to grade 3 later by the principal investigator, and was therefore not captured as a DLT at the time; both patients continued on the trial.

### Cardiac toxicity

**Figure 1:**
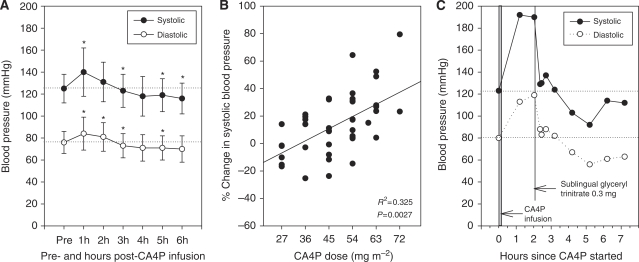
(**A**) Systolic and diastolic blood pressure (mean±s.d.) before and every hour for 6 h after combretastatin A4 phosphate (CA4P) infusion in all 46 patients during the first cycle. ^*^P<0.05 *vs* pretreatment measurement. *n*=46 patients. (**B**) Relationship between CA4P dose (mg m^−2^) and systolic blood pressure changes (in percent) 1 h after CA4P infusion during the first cycle with fitted regression line. *n*=46 patients. (**C**) Blood pressure changes in a patient with hypertension after CA4P, responding to 0.3 mg sublingual glyceryl trinitrate.

In all, 12 of the 46 patients (26%) experienced hypertension, most were CTCAE grade 1 (Table 4). Dose-limiting toxicity of grade 3 hypertension, which was asymptomatic, was seen in one patient at 72 mg m^−2^ CA4P. In general, both systolic and diastolic blood pressures were highest at 1 h, followed by slight hypotension 4 h after CA4P infusion (Figure 1A). Changes in both systolic and diastolic blood pressures correlate significantly with the CA4P dose during the first cycle (Figure 1B, data shown for systolic blood pressure only). Ten patients were already on antihypertensive medication at study inclusion. Eight of these patients showed at least one episode of hypertension during the study. For comparison, only 4 of the 36 patients not taking antihypertensive medication developed at least one episode of hypertension during the study. Only four patients needed treatment for hypertension related to study medication, of whom two were already on antihypertensive medication. Study guidelines initially advised hydralazine and clonidine to be given when systolic blood pressure exceeded 180 mmHg, but this was later amended to glyceryl trinitrate (GTN). Only the last patient in the study experienced hypertension who needed treatment with GTN sublingually (Figure 1C).

QTc prolongations >450 ms were seen in 15 patients (33%) of mostly CTCAE grade 1, and in three patients with grade 2, the longest interval being 480 ms. Patients from all three treatment arms were affected. Two of the patients with QTc prolongations were on antihypertensive medication at study entry. Grade 1 tachycardia was seen in 11% of patients. No cardiac enzyme measurements were taken as none of the patients had clinical symptoms of myocardial ischemia. The only patient with a known history of ischemic heart disease did not experience hypertension or QTc prolongation.

### Pharmacokinetics

Complete plasma data were available for 12 patients for estimation of PK variables for carboplatin (Table 5), and for 31 patients for estimation of PK variables for paclitaxel. Pharmacokinetics could not be estimated for all patients because of sampling problems. The differences between calculated and measured carboplatin AUC did not differ significantly between the different CA4P dose groups, or for the group also receiving paclitaxel, indicating no pharmacological interaction between these drugs using this regimen. Table 5 also shows nadir blood counts demonstrating no relationship with measured carboplatin AUC. A total of 27 patients who received paclitaxel 175 mg m^−2^ had the AUC of paclitaxel measured, which varied from 463 to 4709 mg min ml^−1^, with no significant relationship to the dose of CA4P, indicating no interaction between these drugs using this regimen (data not shown).

### Response

#### Patients with ovarian cancer

Seven of 18 (39%) patients with epithelial ovarian cancer, primary peritoneal carcinoma, or cancer of the fallopian tube had a response according to RECIST and/or GCIG CA-125 criteria. Of them, one had a confirmed and two had an unconfirmed partial remission (PR) according to RECIST, and a response according to GCIG CA-125 criteria, and four had a response according to GCIG CA-125 criteria only.

#### Patients with non-ovarian cancer

Three of 30 (10%) patients with non-ovarian cancer showed PR according to RECIST: one in trial arm 2 with extensive small-cell lung cancer, progressing to 2 months after previous response to carboplatin, etoposide, and thalidomide within a phase III trial; another in trial arm 2 with an adenocarcinoma of the oesophagus–gastric junction, for relapse after surgical resection and adjuvant epirubicin/cisplatin/capectabine, followed by second-line mitomycin C chemotherapy; and a third in trial arm 3 with metastatic melanoma of the skin progressing during first-line trial therapy with dacarbazine and sorafenib.

## Discussion

This phase Ib trial was planned because of the severe myelosuppression observed when combining CA4P with carboplatin in a previous study and the concerns about combining the two different tubulin-binding agents, combretastatin A4 phosphate and paclitaxel. It was the concern about the possibility of CA4P enhancing haematological and neurotoxicity that led us to perform this trial rather than performing a phase II trial with a cohort of patients treated with a lower dose of CA4P, as was the approach followed in the trial of another VDA, DMXAA (Rustin *et al*, 2003b).

However, this trial has shown that it is safe to combine doses of CA4P that have been shown to reduce tumour blood flow (Anderson *et al*, 2003; Galbraith *et al*, 2003; Stevenson *et al*, 2003) with doses of carboplatin and paclitaxel that are considered standard therapy. In a previous study, CA4P 27–36 mg m^−2^ was given 1 h after carboplatin AUC 4 or 5, resulting in 56% (9 of 16) of patients suffering grade 3 or 4 thrombocytopenia (Bilenker *et al*, 2005). In this study, grade 3 or 4 thrombocytopenia was seen only in 4% of patients with similar doses of carboplatin but with doses of CA4P up to 72 mg m^−2^. Administering carboplatin at least 20 h after CA4P has dramatically reduced myelosuppression. The degree of grade 3 or 4 myelosuppression with the triplet was lower than that observed previously without CA4P (Vasey *et al*, 2004).

As ataxia and motor neuropathy were DLTs in the first phase I trial of CA4P at 114 mg m^−2^ (Rustin *et al*, 2003a), the starting dose when combined with the neurotoxic drug paclitaxel was reduced to 27 mg m^−2^. In this study, neurotoxicity was seen in the form of sensory neuropathy in 54% of patients, mostly of CTCAE grade 1 (41% of patients) and probably more related to paclitaxel than to CA4P. Motor neuropathy, ataxia, and dysphasia were observed in a total of six patients (13%) at doses between 45 and 72 mg m^−2^ CA4P and were of short duration, lasting a couple of minutes to a few hours.

Patients who already took medication to control arterial hypertension at the time of study entry were more likely to experience hypertension after CA4P infusion. One patient received GTN for the treatment of CTCAE grade 1 hypertension, which proved to be highly effective in quickly normalising blood pressure, but we now recommend giving GTN as a dermal patch to reduce the side effects of sublingual GTN. The notion that nitrates and calcium channel blockers such as amlodipine are highly effective has recently been corroborated by a study in rats (Ke *et al*, 2009). There remains a concern that hypertension induced by CA4P could cause myocardial or cerebral damage, especially as many elderly patients with cancer have preexisting cardiovascular disease. As we previously demonstrated that CA4P efficiency is dose dependent (Rustin *et al*, 2003a), and have evidence from this study that hypertension also follows a dose-dependent pattern, we have introduced the prophylactic use of amlodipine in current protocols for the ongoing clinical development of combretastatin A1 phosphate.

Pharmacokinetics showed no evidence of pharmacological interaction between carboplatin or paclitaxel and CA4P. Dose-limiting toxicities were CTCAE grade 3 ataxia and CTCAE grade 3 arterial hypertension at 72 mg m^−2^ CA4P. Thus, the recommended dose for phase II trials is 63 mg m^−2^ CA4P, combined with carboplatin AUC 5 (based on GFR measured by EDTA clearance), and paclitaxel 175 mg m^−2^.

It is encouraging that responses were observed in a variety of tumour types, including ovarian cancer (7 of 18 patients responding), small-cell lung cancer, adenocarcinoma of the oesophagus–gastric junction, and malignant melanoma (three patients with PR), demonstrating response in a total of 22% of all patients (including two patients with unconfirmed response). The high response rate in ovarian cancer demonstrates further evidence that this disease is responsive to vascular strategies, and in consequence, a phase II trial in platinum-resistant ovarian cancer has been completed.

## Figures and Tables

**Table 1:** Dose-escalation scheme: treatment cohorts with number of patients treated and courses given

**Trial arms**	**Arm 1: CA4P/Carboplatin**					**Arm 2: CA4P/Paclitaxel**					**Arm 3: CA4P/Carboplatin/Paclitaxel**		
**Cohort**	**1**	**2**	**3**	**4**	**5**	**1**	**2**	**3**	**4**	**5**	**1**	**2**	**3**
CA4P dose (mg m^−2^)	36	45	45	60	54	27	27	36	45	54	54	63	72
Carboplatin AUC	4	4	5	5	5	—	—	—	—	—	5	5	5
Paclitaxel dose (mg m^−2^)	—	—	—	—	—	135	175	175	175	175	175	175	175
Number of patients	3	3	3	1	5	4	3	3	4	5	4	6	2
Total number of cycles	14	8	11	1	21	16	11	10	11	20	23	27	6

Abbreviations: AUC=area under the concentration curve; CA4P=combretastatin A4 phosphate.

**Table 2:** Patient characteristics (*n*=46)

**Characteristics**	**No. of patients**
Assessable for response	46
	
*Gender*	
Male	18
Female	28
	
*Age, years*	
Median	57
Range	24–77
	
*WHO performance status*	
0	13
1	29
2	4
	
*Prior therapy*	
Chemotherapy	44
Radiotherapy	7
Hormonal or biological therapy	13
	
*Tumour type*	
Ovary	16
Melanoma	6
Colorectal	5
Kidney	5
Lung	3
Oesophagus	2
Thyroid	2
Gastrointestinal stroma tumour	1
Neuroendocrine tumour	1
Cholangiocarcinoma	1
Leiomyosarcoma of the uterus	1
Testis	1
Primary peritoneal carcinoma	1
Fallopian tube	1

Abbreviation: WHO=World Health Organization.

**Table 3:** Worst haematological toxicity per patient (all cycles)

	**Number of patients**		
	**Carboplatin arm**	**Paclitaxel arm**	**Triplet arm**
*CTCAE grade 1*			
Anaemia	7	9	5
Lymphopenia	3	2	2
Neutropenia	2	—	—
Thrombocytopenia	4	2	7
			
*CTCAE grade 2*			
Anaemia	6	5	6
Lymphopenia	—	—	—
Neutropenia	2	6	2
Thrombocytopenia	1	—	—
			
*CTCAE grade 3–4*			
Anaemia	—	1	1
Lymphopenia	2	3	1
Neutropenia	—	—	8
Thrombocytopenia	—	1	1

Abbreviation: CTCAE=common terminology criteria for adverse events.

**Table 4:** Worst non-haematological drug-related toxicity per patients (all cycles)

**Treatment arm**	**Number of patients**								
	**Carboplatin arm**			**Paclitaxel arm**			**Triplet arm**		
**CTCAE grade Toxicity**	**1**	**2**	**3**	**1**	**2**	**3**	**1**	**2**	**3**
Fatigue	4	5	3	5	7	—	3	5	—
Pain	6	4	2	2	8	2	1	5	0
Sensory neuropathy	6	—	1	8	1	1	5	3	—
Alopecia	—	—	—	2	12	—	1	7	—
Nausea	4	5	—	4	2	—	3	4	—
Pain – headache	1	5	1	3	1	—	1	4	—
QTc prolongation	3	—	—	7	2	—	2	1	—
Vomiting	3	2	1	3	3	—	—	2	—
Hypertension	3	2	—	1	2	—	3	—	1
Constipation	—	4	—	1	4	—	—	2	—
Diarrhoea	2	1		3	1	1	—	2	3
Pyrexia	3	—	—	5	2	—	1	—	—
Flushing	3	1	—	1	—	—	2	1	—
Hypotension	—	1	—	1	3	—	1	2	—
Itching	3	—	—	1	—	—	4	—	—
Anorexia	1	1	—	—	—	—	4	1	—
Infection	—	1	—	—	1	2	2	1	—
Myalgia	1	—	—	3	—	—	2	1	—
Neurological: muzzy head	2	—	—	2	—	—	1	—	—
Oedema	2	1	—	1	1	—	—	—	—
Perianal itching	3	—	—	1	—	—	1	—	—
Rash	—	1	—	1	1	2	—	—	—
Stomatitis	1	—	—	1	1	—	2	—	—
Tachycardia	1	—	—	2	—	—	2	—	—
Agitation	2	—	1	1	—	—	—	—	—
Cough	—	1	—	1	—	1	—	—	—
Motor neuropathy	—	1	1	—	—	1	—	—	—
Allergy	—	—	1^a^	—	—	1	1	—	—
Ataxia	—	—	—	—	—	—	—	1	1
Dysphasia	—	1	1	—	—	—	—	—	—
Dehydration	—	—	—	—	—	1	—	—	—
Incontinence	—	—	1	—	—	—	—	—	—
Sepsis	—	—	1	—	—	—	—	—	—

Abbreviation: CTCAE=common terminology criteria for adverse events.

a Grade 4 toxicity.

**Table 5:** Carboplatin pharmacokinetics and blood count nadirs

**Patient no.**	**Carboplatin AUC calculated**	**CA4P dose (mg m^−2^)**	**Paclitaxel dose (mg m^−2^)**	**Carboplatin AUC measured (mg min ml^−1^)**	**Carboplatin *C*_max_ (ng ml^−1^)**	**Haemoglobin nadir (g per 100 ml)**	**Neutrophil nadir ( × 10^3^ μl^−1^)**	**Platelet nadir ( × 10^3^ μl^−1^)**
10	4	45	—	4.03	24700	12.7	2.31	203
12	4	45	—	4.08	24800	8.5	5.40	302
13	4	45	—	8.66	105000	12.0	2.26	147
14	5	45	—	5.79	41100	11.0	2.72	103
15	5	45	—	5.55	31900	8.6	3.32	175
17	5	45	—	5.24	41300	10.0	3.31	165
21	5	54	—	6.71	31200	10.1	2.85	106
23	5	54	—	5.38	33200	10.6	2.51	215
32	5	54	—	4.60	21000	10.1	1.78	172
34	5	54	—	3.27	11500	12.4	1.60	196
39	5	63	175	3.98	15700	9.7	0.63	323
41	5	63	175	3.85	21100	10.5	0.68	137

Abbreviations: AUC=area under the concentration curve; CA4P=combretastatin A4 phosphate.
